# Hyperbaric oxygen therapy as an adjunctive treatment for COVID-19-associated mucormycosis: a report of two cases

**DOI:** 10.17843/rpmesp.2023.401.11980

**Published:** 2023-03-22

**Authors:** Víctor Valencia-Caballero, José Cachay-Diaz, José Huamán-Muñante, Lourdes Romaní-Montoro, Norka Vásquez-Zevallos, César Carozzi-Calvo, Cesar Chian-García, Hugo Hernández-Chávez, Cristhian Agustín-Paredes, Esteban Vergara-de la Rosa

**Affiliations:** 1 Internal Medicine Department, Hospital Nacional Arzobispo Loayza, Lima, Peru. Internal Medicine Department Hospital Nacional Arzobispo Loayza Lima Peru; 2 Otorhinolaryngology Department, Hospital Nacional Arzobispo Loayza, Lima, Peru. Otorhinolaryngology Department Hospital Nacional Arzobispo Loayza Lima Peru; 3 Hyperbaric Medicine Center, Lima, Peru. Hyperbaric Medicine Center Lima Peru; 4 Anatomic Pathology Department, Hospital Nacional Arzobispo Loayza, Lima, Peru. Anatomic Pathology Department Hospital Nacional Arzobispo Loayza Lima Peru; 5 Otorhinolaryngology Department, Hospital Regional Docente de Trujillo, Trujillo, Peru. Otorhinolaryngology Department Hospital Regional Docente de Trujillo Trujillo Peru

**Keywords:** Mucormycosis, Hyperbaric Oxygenation, COVID-19, Diabetes Mellitus, Amphotericin B, Natural Orifice Endoscopic Surgery

## Abstract

We present the first two cases reported in Peru of the use of adjuvant hyperbaric oxygen therapy (HBOT) in patients with COVID-19-associated mucormycosis (CAM). The first case is a 41-year-old woman, with pain in the left side of the face and palatine region with purulent rhinorrhea for a month. Only an oroantral fistula was found during physical examination. The second case is a 35-year-old male, with decreased left visual acuity and palatal pain with a fistula, draining purulent secretion for four months. Both patients have history of diabetes, had moderate COVID-19 four months prior to admission, and received corticosteroid therapy for this diagnosis. Tomographic evaluation of both patients showed involvement of the maxillary sinus and surrounding bone tissue; both received diagnostic and therapeutic nasal endoscopy for debridement. Histological analysis showed that the samples were compatible with mucormycosis. The patients underwent debridement and were treated with amphotericin B deoxycholate; however, they presented torpid evolution. Then, HBOT was added and the patients showed an evident improvement after four weeks of treatment with subsequent controls without the presence of mucormycosis. We highlight the favorable evolution of these patients while receiving HBOT as treatment for a disease with high morbimortality, which emerged during the pandemic.

## INTRODUCTION

Mucormycosis is an angioinvasive fungal infection of the genus Mucorales, that evolves rapidly and progressively causing tissue necrosis ^1^^-^^3^. This infection is mainly spread by the airborne route. Mucormycosis mainly affects people with diabetes and immunocompromised patients ^4^^-^^6^. Rhino-orbito-cerebral mucormycosis (ROCM) is the most frequent type ^1^^,^^4^. Currently, cases associated with diabetes mellitus (T2DM) and SARS-CoV-2 virus have increased ^5^^-^^7^. Histopathology and/or culture establish the definitive diagnosis ^1^^-^^4^. Standard treatment (ST) is medical-surgical (intravenous amphotericin and surgery), and patient survival improves with early diagnosis and treatment ^3^^,^^8^^,^^9^. At the time of this report, adjuvant hyperbaric oxygen therapy (HBOT) has been used as treatment in patients with mucormycosis without COVID-19 ^10^^-^^12^. 

We report the first two cases, in Peru, of COVID-19-associated mucormycosis (CAM), where HBOT was added to standard treatment, with good response. Previous studies have described favorable results with the use of HBOT ^10^^-^^12^.

## CASE REPORT

### Case 1

**Figure 1:**
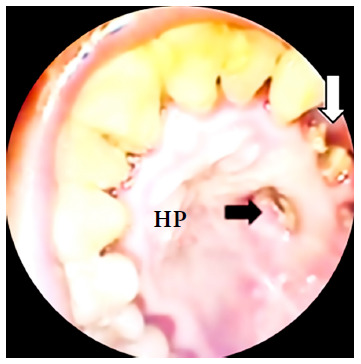
Black arrow: oroantral fistula in the hard palate, white arrow: loss of teeth. HP: hard palate.

We present the case of a 41-year-old woman from Lima with mild COVID-19 four months before admission and diagnosed with mucormycosis by dental socket biopsy. She had moderate pain in the left hemiface, upper dental arch, hard palate and ipsilateral rhinorrhea for one month. The patient had a history of T2DM and arterial hypertension under treatment. On physical examination, she presented left oroantral fistula with purulent discharge (Figure 1). Laboratory tests showed glucose levels of 85 mg/dl, glycosylated hemoglobin (HbA1c) of 9.26 and a negative HIV test. The diagnosis of mucormycosis was confirmed by histopathology with hematoxylin-eosin (HE) staining (Figure 4A).

**Figure 2:**
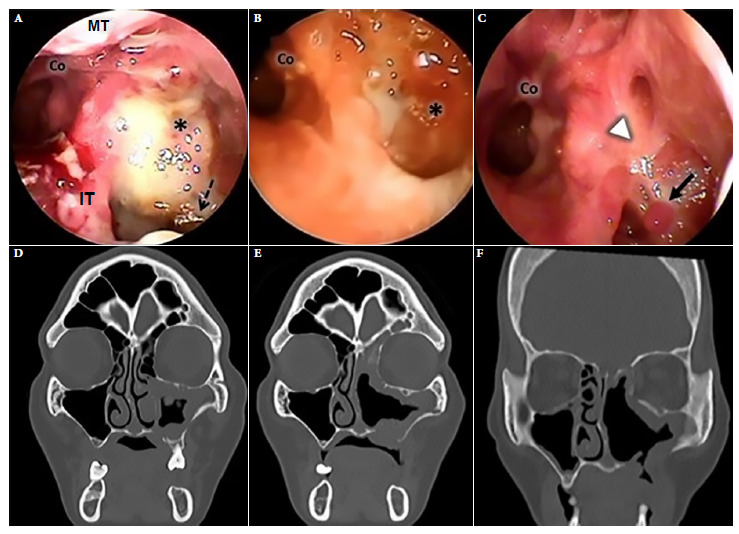
Case 1. Left nasal endoscopy. (A) Admission endoscopy (B) Endoscopy during standard treatment. (C) Endoscopy after 30 sessions of treatment with adjuvant hyperbaric oxygen. Facial CT scan. (D) On admission, hypertrophy of the mucosa of the left maxillary sinus, bone lysis of its medial and inferior wall and palatine bone. Obstruction of the left ostiomeatal complex. (E) CT scan during standard treatment. (F) Tomographic control after 30 sessions of treatment with adjuvant hyperbaric oxygen, shows improvement of the nasal mucosa and oroantral fistula. Asterisk: posterior wall of maxillary sinus, Co: choana, S: septum, IT: inferior turbinate, MT: middle turbinate; dashed black arrow: devitalized bone tissue, white arrowhead: scar mucosa, continuous black arrow: granulation tissue.

Nasal endoscopic and tomographic evaluation happened at three points in time: at the beginning of conventional treatment, 35 days after initiation of ST and after 30 sessions of HBOT (Figure 2). Amphotericin B deoxycholate 50 mg/day was administered for 50 days, in addition to intravenous ciprofloxacin. Nasal endoscopy evidenced necrosis of bone tissue in the posterior wall of the left maxillary sinus in addition to purulent discharge at baseline ST (Figure 2A). The inferior and middle turbinate were absent at day 35 of ST, with polypoid-like tissue formation and persistent discharge (Figure 2B). Thirty sessions of HBOT were added after day 35 of ST, after which the sinus mucosa improved with scar-like tissue and granulation without purulent content or necrosis at endoscopy (Figure 2C).

Computed tomography (CT) of the paranasal sinuses on admission showed findings compatible with mucormycosis in the left maxillary sinus and palatine bone (Figure 2D). Persistent thickening of the sinus mucosa was evidenced on day 35 of ST. After 30 sessions of HBOT, the tomographic images improved significantly (Figures 2D, 2E and 2F).

Endoscopic sinus surgery (ESS) for debridement was performed twice. But endoscopic-tomographic evaluation after ST showed that the disease persisted (Figures 2B, 2E). However, after the addition of 30 sessions of HBOT the patient improved. Each session lasted 60 min per day, at 2.8 atmospheres with 100% oxygen, on a daily basis. No adverse events were reported. The patient was followed-up for 6 months, with no recurrence. Informed consent was obtained for the preparation and publication of this report.

### Case 2

**Figure 3:**
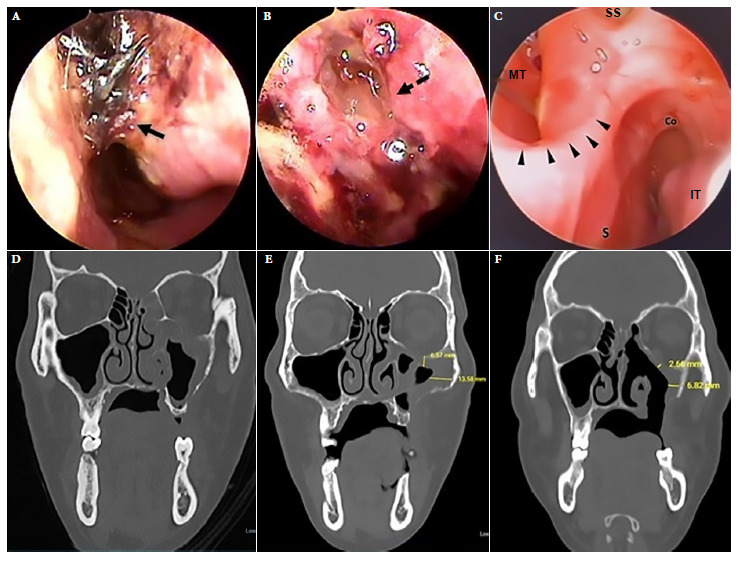
Case 2. Left nasal endoscopy. (A) Endoscopy on admission. (B) Endoscopy during standard treatment. (C) Endoscopy after 30 sessions of treatment with adjuvant hyperbaric oxygen. (D) Facial CT scan. On admission, mucosal hypertrophy of the left maxillary sinus, occupation of anterior ethmoid cells and dehiscence of the orbital floor. (E) CT scan during standard treatment. (F) Tomographic control after 30 sessions of treatment with adjuvant hyperbaric oxygen showing decreased mucosal thickening and a left oroantral fistula. Continuous black arrow: necrotic crusts, dashed black arrow: cerebrospinal fluid fistula, black arrowheads: septal perforation margin. S: septum, IT: inferior turbinate, MT: middle turbinate, SS: sphenoid sinus, Co: choana.

**Figure 4:**
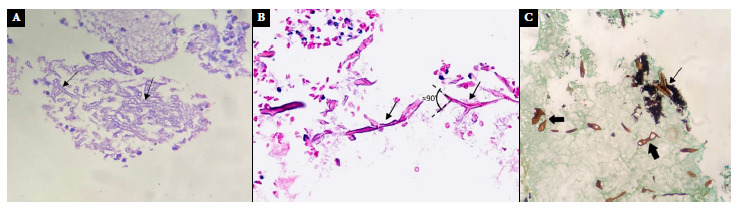
(A) case 1, (B) case 2. Nasosinusal mucosa with Hematoxylin-Eosin staining. (C) case 2. Nasal mucosa with Grocott’s methenamine silver stain at 40X showing wide ribbon-like pauciseptate hyphae, some of them deformed (thick black arrow), others with branching (thin black arrow) and areas of necrosis.

We present the case of a 35-year-old male, referred from another hospital, diagnosed with CAM by histopathology who had a positive molecular test for SARS-CoV-2 virus. He presented progressive decrease of visual acuity in the left eye four months prior to admission and moderate stabbing pain in the hard palate. He had a history of T2DM and right hemiparesis as a sequel to a previous ischemic cerebrovascular disease. Physical examination showed partial dental loss and left oroantral fistula of 1.5 cm diameter in the middle third of the ipsilateral hard palate, left peripheral facial paralysis, absent corneal reflex and ipsilateral ophthalmoplegia. Laboratory tests showed blood glucose at 125 mg/dL, HbA1c at 5.6 and a negative HIV test. Nasal endoscopy on admission showed necrotic crusts (Figure 3A). A CT of the paranasal sinuses showed signs compatible with mucormycosis in the maxillary sinus, ethmoid and left sphenoid sinus (Figure 3D). Histologic examination of the left maxillary sinus mucosa with hematoxylin eosin and Grocott-Gomori methenamine silver staining showed mucor hyphae (Figures 4B and 4C), which confirmed the diagnosis of mucormycosis.

Debridement was carried out during the ESS; biopsy samples were also collected during surgery. Disease progression was evidenced by tomographic images. Nasal endoscopy revealed a cerebrospinal fluid fistula in the roof of the left anterior portion of the ethmoid bone as well as necrosis of the nasal attic mucosa (Figure 3B). Given the torpid clinical evolution at day 50 of ST, HBOT was added for 60 min/day, at 2.8 atmospheres with 100% oxygen for 30 sessions on a daily basis. No adverse events were reported. Endoscopic and tomographic improvement was evident after HBOT (Figures 3C, 3F). Amphotericin B deoxycholate was administered for 71 days. The patient was followed up for 6 months, with no recurrence. Informed consent was obtained for the preparation and publication of this report.

## DISCUSSION

Mucormycosis is an infrequent, multisystemic, rapidly progressive, rarely suspected mycosis with high morbidity and mortality rates ^1^^-^^3^. The etiological agents belong to the order of mucorales whose genera include *Absidia*, *Cuninghamdla*, *Rhizomucor* and *Rhizopus*. The latter is responsible for 70% of cases ^2^^-^^5^. Spores, their infectious agents, are found in all environments, particularly in unsanitary conditions ^2^^,^^6^. Inhalation is the most frequent mode of transmission, followed by the cutaneous and digestive routes ^1^^,^^4^^,^^7^. In addition, this type of fungus is angioinvasive and affects endothelial cells causing thrombosis with subsequent necrosis and hemorrhage ^4^. T2DM is the most frequent risk factor ^1^^,^^2^^,^^5^^,^^6^^,^^8^, it favors the development of mucorales inhibiting phagocytosis and neutrophil chemotaxis ^2^^,^^8^, increasing free iron, and facilitating endothelial endocytosis ^2^^,^^4^. It rarely affects immunocompetent patients ^2^.

Worldwide, the cases of mucormycosis increased during the COVID-19 pandemic ^2^^,^^5^^-^^8^. The risk factors for this condition are: T2DM, whose prevalence is similar to the population with mucormycosis without COVID-19 ^8^; the indiscriminate use of corticosteroids, which is an important risk factor compared to the population with mucormycosis without COVID-19 ^4^^-^^6^^,^^8^^,^^9^ and the SARS-CoV-2 virus as a risk factor that probably plays a role during etiopathogenesis ^6^. In most cases of CAM, COVID-19 was mild to moderate ^9^.

ROCM, independently of its association with COVID-19, is the most frequent clinical form, particularly in patients with T2DM ^1^^-^^3^^,^^5^, who present facial pain, fever and rhinorrhea. It affects the palate by causing fistulas, and the orbit by causing palpebral ptosis, oculomotor palsy, proptosis and even blindness. Several of these characteristics were found in the two cases reported in this article. The affection of consciousness is evidence of central nervous system (CNS) involvement ^1^^,^^3^.

CT is the test of choice, which identifies paranasal sinus alteration (mucosal thickening and adjacent bone erosions) and orbital involvement ^3^^,^^9^. On the other hand, MRI is recommended to rule out CNS involvement ^3^, while nasal endoscopy is essential for surgical treatment and sampling.

Diagnosis is confirmed by: i) histopathology: with HE staining, periodic acid-Schiff (PAS) staining or Grocott-Gomori’s methenamine silver stain, which were used in the two cases presented (Figure 4); and ii) microbiology: with fresh tissue examination and Giemsa staining ^7^. Polymerase chain reaction (PCR) techniques are more specific, but not very accessible, while culture allows identification of the species ^3^^,^^7^.

Treatment is based on early diagnosis, administration of antifungals, surgical removal of infected tissue, obtaining a good response in 71% of cases ^10^; and restoration of the immune system in some cases ^1^^,^^3^. Amphotericin B is the antifungal of choice in its three forms: liposomal (5-10mg/kg), lipid complex and deoxycholate, the latter being potentially nephrotoxic ^1^^,^^3^^,^^10^. Imidazoles such as posaconazole and isavuconazole are complementary or alternative therapies in case of intolerance or poor response to amphotericin ^3^^,^^10^. The adequate length of treatment has not been stablished yet. Treatment has been reported to last from weeks to years averaging 3 to 6 months, until clinical and tomographic reversion and absence of mucorales is achieved ^3^.

Mortality ranges between 32 and 80% in patients without COVID-19 diagnosis ^3^^,^^7^^,^^10^, depending on the state of immunity (cellular and neutropenia), age, associated malignancy and location. If there is sinus and CNS involvement, mortality reaches 30% and 80%, respectively ^3^. On the other hand, according to the information consulted, mortality in patients with CAM is variable; some studies report ranges from 9.8% ^8^ to 30% ^9^. These differences could depend on several factors: presence of associated comorbidities, level and speed of healthcare response, type of SARS-CoV-2 variant, country ^2^ and extent of the disease ^3^. Early diagnosis and treatment improve prognosis. Due to the significant morbimortality, the literature reports some adjuvant therapies to ST, among them, HBOT ^1^^,^^11^^-^^13^.

HBOT has the following effects on infectious diseases: a) it improves the phagocytic activity of oxygen-dependent leukocytes ^11^^,^^14^^,^^15^; b) it corrects lactic acidosis by favoring the oxidative action of amphotericin B ^11^^,^^14^; and c) it favors tissue repair ^10^^,^^11^^,^^15^ by activation of fibroblasts and neovascularization due to an increase in growth factors ^9^^-^^11^^,^^15^. Therefore, it has been used in cases of necrotizing soft tissue infections such as necrotizing fasciitis, gas gangrene, chronic refractory osteomyelitis and diabetic foot ulcers ^11^^,^^15^. HBOT is usually well tolerated, with low risk of adverse events ^9^.

There are few reports of HBOT being used in cases of mucormycosis, these include case series, but no randomized clinical trials. In 1988, Berrylin reported the first case series where HBOT was used, the study included 6 cases, 4 of which were successful ^12^. Kontoyannis reported in 2005, in a series of 28 cases, a survival rate of 94% in diabetic patients with mucormycosis; 75% of the patients had ROCM, with a mean number of sessions of 22 ^13^. Valente *et al*. reported successful treatment in 6 of 7 cases in 2021 ^15^.

One of the cases we report was a female who presented the sinus form and the other one was a male with the rhino-orbital form, HBOT was added in both cases due to the lack of response after almost two months of ST (amphotericin B deoxycholate and surgical debridement). One of the patients presented renal toxicity (creatinine: 2.3 mg/dL). When HBOT was added, both patients showed evident clinical-tomographic and endoscopic improvement, with negative samples for fungi and normal controls six months after discharge. Thus, this would be the first report of the use of HBOT in patients with CAM.

Although, in both cases, HBOT was administered after 50 days of standard treatment, it is likely that its addition to conventional treatment could improve the patient’s prognosis. In addition, although both patients were followed-up for six months, we believe it is necessary to increase the minimum follow-up time to one year, in order to achieve greater reliability in the sustained therapeutic response over time.

One of the limitations of this report is the lack of availability of HBOT in the hospital centers of the Peruvian Ministry of Health, its high cost and the absence of a health insurance that assumes the expenses of its application. The cost of 30 sessions of HBOT in Peru is around 1500 US dollars. In order to carry out this study, it was necessary to request the collaboration of a private institution, which provided HBOT for both patients. We highlight the possibility that the clinical improvement in both cases may be independent of the application of HBOT. For this reason, studies with a higher level of evidence are necessary to understand the specific benefit of HBOT in the treatment of mucormycosis.

In conclusion, CAM emerged during the COVID-19 pandemic and currently shows significant morbidity and mortality rates; early management is essential to control the infection. HBOT as an adjuvant treatment could shorten the length of treatment and hospital stay, decreasing the exposure to nephrotoxicity and reducing morbidity. Additionally, we highlight the need for new studies that can provide a higher level of evidence.
