# Supplementary material for: Hyperbaric oxygen therapy as an adjunctive treatment for COVID-19-associated mucormycosis: a report of two cases
Source: Rev Peru Med Exp Salud Publica. 2023 Mar 22;40(1):105–10. doi: 10.17843/rpmesp.2023.401.11980 (PMC10953642; doi:10.17843/rpmesp.2023.401.11980)
Supplement: Translation

# Translation: Oxigenoterapia hiperbárica como tratamiento complementario en mucormicosis asociada a COVID-19: a propósito de dos casos

**Authors:** Víctor Valencia-Caballero, José Cachay-Diaz, José Huamán-Muñante, Lourdes Romaní-Montoro, Norka Vásquez-Zevallos, César Carozzi-Calvo, Cesar Chian-García, Hugo Hernández-Chávez, Cristhian Agustín-Paredes, Esteban Vergara-de la Rosa

**Affiliations:** 1 Servicio de Medicina Interna, Hospital Nacional Arzobispo Loayza, Lima, Perú.; 2 Servicio de Otorrinolaringología, Hospital Nacional Arzobispo Loayza, Lima, Perú.; 3 Centro de Medicina Hiperbárica, Lima, Perú.; 4 Servicio de Anatomía Patológica, Hospital Nacional Arzobispo Loayza, Lima, Perú.; 5 Servicio de Otorrinolaringología, Hospital Regional Docente de Trujillo, Trujillo, Perú.

**Keywords:** Mucormicosis, Oxigenoterapia Hiperbárica, COVID-19, Diabetes Mellitus, Anfotericina B, Cirugía Endoscópica por Orificios Naturales

## Abstract

Reportamos los dos primeros casos, en Perú, sobre el uso del tratamiento con oxigeno hiperbárico coadyuvante (TOHC) en pacientes con mucormicosis asociado a COVID-19 (MAC). El primer caso es una mujer de 41 años, con dolor en hemicara y región palatina izquierdas con rinorrea purulenta de un mes de evolución. Al examen físico, solo evidencia fístula oroantral. El segundo caso se trata de un varón de 35 años, con disminución de agudeza visual izquierda y dolor palatino con fístula que drena secreción purulenta de cuatro meses de evolución. Ambos pacientes tienen el antecedente de diabetes, tuvieron COVID-19 moderado cuatro meses antes del ingreso, y recibieron corticoterapia por este diagnóstico. Ambos pacientes tuvieron una evaluación tomográfica que mostró compromiso del seno maxilar y tejido óseo circundante, con endoscopia nasal diagnóstica y terapéutica para desbridamiento. Se obtuvieron muestras compatibles con mucormicosis en estudio histológico. Los pacientes fueron tratados con limpieza quirúrgica y anfotericina B desoxicolato, sin embargo, presentaron evoluciones tórpidas. Por lo tanto, se adicionó la TOHC y mostraron una mejoría evidente tras cuatro semanas de tratamiento con controles subsiguientes sin presencia de mucormicosis. Resaltamos la evolución de estos pacientes con TOHC, en una enfermedad con importante morbimortalidad, que ha sido emergente durante la pandemia.

## INTRODUCCIÓN

La mucormicosis es una infección micótica del género mucorales, de carácter angioinvasiva con evolución rápida y progresiva que ocasiona necrosis tisular ^1^^-^^3^. La vía inhalatoria es la forma de ingreso más frecuente. Afecta principalmente a diabéticos e inmunocomprometidos ^4^^-^^6^. El tipo de presentación más frecuente es la rino-orbito-cerebral (ROC) ^1^^,^^4^. Actualmente, se ha registrado un incremento de casos asociados a diabetes mellitus (DM2) y el virus SARS-CoV-2 ^5^^-^^7^. La histopatología y/o cultivo establecen el diagnóstico definitivo ^1^^-^^4^. El tratamiento convencional (TC) es médico-quirúrgico (anfotericina endovenosa y cirugía), cuando se establece el diagnóstico y se administra un tratamiento precoz, mejora la sobrevida del paciente ^3^^,^^8^^,^^9^. El tratamiento con oxigeno hiperbárico coadyuvante (TOHC), al momento del presente reporte, ha sido informado como tratamiento en pacientes con Mucormicosis sin COVID-19 ^10^^-^^12^. 

Reportamos los dos primeros casos, en Perú, de mucormicosis asociado a COVID-19 (MAC), donde se adicionó la TOHC, además del tratamiento estándar, con buena respuesta. Estudios previos han descrito resultados favorables con el uso de la TOHC ^10^^-^^12^.

## REPORTE DE CASO

### Caso 1

Mujer de 41 años, procedente de Lima, con COVID-19 leve cuatro meses antes de ingreso y diagnóstico de mucormicosis mediante biopsia de alveolo dentario. Presentó dolor moderado en hemicara izquierda, arcada dentaria superior, paladar duro y rinorrea ipsilateral de un mes de evolución. La paciente tiene historia de DM2 e hipertensión arterial en tratamiento. Al examen físico, presentó fistula oroantral izquierda con secreción purulenta (Figura 1). Los exámenes laboratoriales muestran niveles de glucosa de 85 mg/dl, hemoglobina glicosilada (HbA1c) de 9,26 y prueba de VIH negativa. El diagnóstico de mucormicosis fue ratificado por histopatología: con tinción hematoxilina-eosina (HE) (Figura 4A).

**Figura 1:** Flecha negra: fístula oroantral a nivel de paladar duro, flecha blanca: perdida de piezas dentarias. PD: paladar duro.

Se reporta la evolución endoscópica nasal y tomográfica en tres momentos: al inicio del tratamiento convencional, a los 35 días de iniciado el TC y después de 30 sesiones de la TOHC (Figura 2). Se administró anfotericina B desoxicolato 50 mg/día durante un periodo total de 50 días, además de ciprofloxacino endovenoso. La endoscopía nasal evidenció necrosis del tejido óseo en la pared posterior del seno maxilar izquierdo además de secreción purulenta al inicio del TC (Figura 2A). Al día 35 del TC, hubo ausencia de cornete inferior y medio con formación de tejido de aspecto polipoide y persistencia de secreciones (Figura 2B). Luego del día 35 del TC, se adicionó 30 sesiones de la TOHC y se evidenció mejoría de las características de la mucosa sinusal con tejido de aspecto cicatricial y focos de granulación sin contenido purulento ni focos de necrosis a la endoscopía (Figura 2C).

**Figura 2:** Caso 1. Endoscopia nasal izquierda. (A) Endoscopia de ingreso**.** (B) Endoscopia durante tratamiento convencional. (C) Endoscopia después de 30 sesiones de tratamiento con oxigeno hiperbárico coadyuvante. Tomografía de macizo facial. (D) Al ingreso, hipertrofia de la mucosa del seno maxilar izquierdo, lisis ósea de su pared medial e inferior y hueso palatino. Obstrucción del complejo osteomeatal izquierdo**. (**E) Tomografía axial computarizada durante el tratamiento convencional. (F) Control tomográfico posterior a 30 sesiones de tratamiento con oxigeno hiperbárico coadyuvante. Muestra mejoría de la mucosa nasal y fístula oroantral. Asterisco: pared posterior de seno maxilar, Co: coana, S: septum, CI: cornete inferior, CM: cornete medio; flecha negra discontinua: tejido óseo desvitalizado, cabeza de flecha blanca: mucosa cicatricial, flecha negra continua: tejido de granulación.

La tomografía axial computarizada (TAC) de senos paranasales al ingreso, evidenció hallazgos compatibles con mucormicosis en el seno maxilar izquierdo y hueso palatino (Figura 2D). Al día 35 del TC, se evidenció persistencia de engrosamiento de mucosa sinusal. Posterior a las 30 sesiones de la TOHC, hubo mejoría tomográfica importante (Figuras 2D, 2E y 2F).

Se realizó cirugía endoscópica nasosinusal (CENS) para desbridamiento en dos oportunidades. La evaluación endoscópica-tomográfica después del TC, mostró persistencia de la enfermedad (Figuras 2B, 2E). Sin embargo, se evidenció una buena respuesta después de la adición de 30 sesiones de la TOHC. Cada sesión tuvo una duración de 60 min por día, a 2,8 atmósferas con oxígeno al 100%, en forma diaria. No se reportó evento adverso alguno. Se realizó el seguimiento clínico de 6 meses, sin presentar recidiva. Se obtuvo el consentimiento informado para la elaboración y publicación del presente reporte.

### Caso 2

Varón de 35 años, referido de otro hospital, con diagnóstico de MAC mediante histopatología y prueba molecular positiva para el virus SARS-CoV-2. Presentó disminución progresiva de agudeza visual izquierda hace cuatro meses y dolor tipo punzante moderado en paladar óseo. Presenta historia de DM2 y hemiparesia derecha como secuela de antecedente de enfermedad cerebrovascular isquémico. El examen físico mostró perdida dentaria parcial y fistula oroantral izquierda de 1,5 cm de diámetro en el tercio medio del paladar duro ipsilateral, parálisis facial periférica izquierda, reflejo corneal ausente y oftalmoplejía ipsilateral. Los exámenes laboratoriales mostraron valores de glicemia de 125 mg/dL, HbA1c de 5,6 y prueba de VIH negativa. La endoscopia nasal al ingreso mostró costras necróticas (Figura 3A). La TAC de senos paranasales presentó hallazgos compatibles con mucormicosis en seno maxilar, etmoidal y esfenoidal izquierdo (Figura 3D). La histopatología de la mucosa de seno maxilar izquierdo evidencio hifas de mucorales con tinciones de hematoxilina eosina y plata metaminada Grocott-Gomori (Figuras 4B y 4C), y confirmó el diagnóstico de mucormicosis.

**Figura 3:** Caso 2. Endoscopia nasal izquierda. (A) Endoscopia de ingreso. (B) Endoscopia durante tratamiento convencional. (C) Endoscopia después de 30 sesiones del tratamiento con oxigeno hiperbárico coadyuvante. **(**D) Tomografía de macizo facial. Al ingreso, hipertrofia de mucosa del seno maxilar izquierdo, ocupación de celdillas etmoidales anteriores y dehiscencia del piso orbitario. (E) Tomografía axial computarizada durante el tratamiento convencional. (F) Control tomográfico posterior a 30 sesiones del tratamiento con oxigeno hiperbárico coadyuvante. Se visualiza engrosamiento mucoso disminuido y fistula oroantral izquierda. Flecha negra continua: costras de aspecto necrótico, flecha negra discontinua: fístula de líquido cefalorraquídeo, cabezas de flechas negras: margen de perforación septal. S: septum, CI: cornete inferior, CM: cornete medio, SE: seno esfenoidal, Co: coana.

Se realizó CENS para desbridamiento y biopsia. Los controles tomográficos mostraron avance de la enfermedad. La endoscopia nasal reveló una fístula de líquido cefalorraquídeo en el techo del etmoides anterior izquierdo y necrosis de la mucosa del ático nasal (Figura 3B). Ante la tórpida evolución clínica al día 50 del TC, se añadió la TOHC durante 60 min/día, a 2,8 atmósferas con oxígeno al 100% por 30 sesiones en forma diaria. No se reportó ningún evento adverso. Luego de la TOHC, se evidenció mejoría endoscópica y tomográfica (Figuras 3C, 3F). Se administró anfotericina B desoxicolato durante un tiempo total de 71 días. Se realizó un seguimiento clínico durante 6 meses, sin presentar recidiva. Se obtuvo el consentimiento informado para la elaboración y publicación del presente reporte.

## DISCUSIÓN

La mucormicosis es una micosis infrecuente, multisistémica, de rápida progresión, poco sospechada y con alta morbimortalidad ^1^^-^^3^. Los agentes etiológicos pertenecen al orden de mucorales cuyos géneros comprenden a *Absidia*, *Cuninghamdla*, *Rizomucor* y *Rhizopus*. Este último es el responsable del 70% de los casos ^2^^-^^5^. Las esporas, sus agentes infecciosos, se encuentran en todos los ambientes, particularmente en condiciones no sanitarias ^2^^,^^6^. Las vías de ingreso en orden de frecuencia son: inhalatoria, cutánea y digestiva ^1^^,^^4^^,^^7^. Además, son angioinvasivos al comprometer a la célula endotelial ocasionando trombosis con necrosis y hemorragia subsecuentes ^4^. La DM2 es el factor de riesgo más frecuente ^1^^,^^2^^,^^5^^,^^6^^,^^8^, esta favorece el desarrollo de mucorales: inhibiendo la fagocitosis y quimiotaxis de neutrófilos ^2^^,^^8^, incrementando el hierro libre, y facilita la endocitosis endotelial ^2^^,^^4^. Raramente afecta a inmunocompetentes ^2^.

La actual pandemia por la COVID-19, muestra incremento mundial de casos de mucormicosis ^2^^,^^5^^-^^8^. Los factores de riesgo de esta entidad son: DM2, cuya prevalencia es similar a la población con mucormicosis sin COVID-19 ^8^, el uso indiscriminado de corticoides, el cual, es un factor de riesgo importante comparado con la población con mucormicosis sin COVID-19 ^4^^-^^6^^,^^8^^,^^9^ y el virus SARS-CoV-2 como un factor de riesgo que, probablemente, cumpla un rol en la etiopatogenia ^6^. En la mayoría de los casos de MAC, la COVID-19 fue de presentación leve a moderado ^9^.

La forma clínica ROC, independientemente de su asociación con la COVID-19, es la más frecuente, sobre todo en pacientes con DM2 ^1^^-^^3^^,^^5^, que presentan dolor facial, fiebre y rinorrea. Puede invadir el paladar originando fistulas, y la órbita ocasionando ptosis palpebral, parálisis de oculomotores, proptosis e incluso producir ceguera. Varios de estos hallazgos se describen en los dos casos del presente reporte. El compromiso de conciencia es evidencia de afectación del sistema nervioso central (SNC) ^1^^,^^3^.

La TAC es el estudio de elección, que identifica la alteración de senos paranasales (engrosamiento de mucosa y erosiones óseas adyacentes) y compromiso orbitario ^3^^,^^9^. Por otro lado, la resonancia magnética se indica para descartar compromiso del SNC ^3^, mientras que la endoscopia nasal es fundamental para el tratamiento quirúrgico y toma de muestras.

El diagnóstico de confirmación es mediante la identificación del hongo por: i) histopatología: con tinciones HE, periodic acid-Schiff (PAS) o tinción de plata metaminada Grocott-Gomori, que fueron métodos diagnósticos de los dos casos presentados (Figura 4); y ii) microbiología: con exámenes en fresco y Giemsa ^7^. Las técnicas de reacción en cadena de la polimerasa (PCR) son más específicas, pero poco accesibles, mientras que el cultivo permite identificar a la especie ^3^^,^^7^.

**Figura 4:** (A) caso 1, (B) caso 2. Mucosa nasosinusal con tinción de Hematoxilina-Eosina. Hifas acintadas con ramificaciones en ángulo de 90° (flecha negra delgada) acompañadas de neutrófilos y material necrótico. (C) caso 2. Mucosa nasal con tinción de plata metaminada de Grocott a 40X. muestra hifas anchas pauciseptadas con apariencia de cinta, algunas de ellas deformadas (flecha negra gruesa), otras con ramificaciones (flecha negra delgada) y áreas de necrosis.

Las bases del tratamiento constituyen: el diagnóstico temprano, administración de antifúngicos, remoción quirúrgica del tejido infectado, obteniéndose buena respuesta en 71% de los casos ^10^; y restauración del sistema inmune en algunos casos ^1^^,^^3^. El antimicótico de elección es anfotericina B en sus tres formas: liposomal (5-10mg/kg), complejo lipídico y desoxicolato, siendo esta última potencialmente nefrotóxica ^1^^,^^3^^,^^10^. Los imidazoles como posaconazol e isavuconazol son terapias complementarias o alternativas de existir intolerancia o mala respuesta a anfotericina ^3^^,^^10^. La duración terapéutica es desconocida, reportan semanas, inclusive años, con media de 3 a 6 meses, hasta conseguir la reversión clínica, tomográfica y ausencia de mucorales ^3^.

La mortalidad oscila entre 32 y 80% en pacientes sin diagnóstico de COVID-19 ^3^^,^^7^^,^^10^, dependiendo del estado de inmunidad (celular y neutropenia), edad, malignidad asociada y localización. Si existe compromiso sinusal y del SNC, la mortalidad alcanza el 30% y 80%, respectivamente ^3^. Por otro lado, la mortalidad en pacientes con MAC es variable, de acuerdo con la información consultada. Algunos reportes van desde 9,8% ^8^ hasta un 30% ^9^. Los diferentes porcentajes podrían ser dependientes de varios factores: presencia de comorbilidades asociadas, nivel y rapidez de respuesta sanitaria, tipo de variante SARS-CoV-2, país de estudio ^2^ y la extensión de la enfermedad ^3^. El diagnóstico e inicio precoz del tratamiento mejoran el pronóstico. Debido a la morbimortalidad importante, la literatura reporta algunas terapias coadyuvantes al TC, entre ellas, la TOHC ^1^^,^^11^^-^^13^.

Los mecanismos de acción de la TOHC en enfermedades infecciosas son: a) mejora la actividad fagocítica de leucocitos dependientes de oxígeno ^11^^,^^14^^,^^15^; b) corrige la acidosis láctica favoreciendo la acción oxidativa de la anfotericina B ^11^^,^^14^; y c) favorece la reparación tisular ^10^^,^^11^^,^^15^ por activación de fibroblastos y neovascularización por incremento de factores de crecimiento ^9^^-^^11^^,^^15^. Por lo tanto, se describe su uso en las infecciones necrotizantes de tejidos blandos como: fascitis necrotizante, gangrena gaseosa, osteomielitis refractaria crónica y ulceras de pie diabético ^11^^,^^15^. La TOHC es usualmente bien tolerada, con bajo riesgo de eventos adversos ^9^.

Hay pocos reportes de TOHC en mucormicosis, limitándose a series de casos, no existiendo ensayos clínicos randomizados. Berrylin presenta la primera serie de casos con la THOC de 6 casos con 4 exitosos en 1988 ^12^. Kontoyannis describe una serie de 28 casos en 2005, donde se reportó una supervivencia de 94% en pacientes diabéticos con mucormicosis, y el 75% de los pacientes tenían presentación ROC, siendo la media del número de sesiones de 22 ^13^. Valente *et al.* reportaron el éxito del tratamiento en 6 de 7 casos en 2021 ^15^.

En nuestros dos casos: una mujer con la forma sinusal y un varón con la forma rinoorbitaria se adicionó la TOHC por falta de respuesta tras casi dos meses del TC (anfotericina B desoxicolato y desbridamiento quirúrgico). Uno de ellos, presentó toxicidad renal (creatinina en 2,3 mg/dL). Al adicionar la TOHC, mostraron evidente mejoría clínica-tomográfica, endoscópica, con muestras negativas para hongos y controles normales luego de seis meses del alta médica. Así, el presente estudio sería el primer reporte de la literatura sobre el uso de la TOHC en pacientes con MAC.

Si bien, en el presente reporte, la TOHC se administró después de 50 días de tratamiento convencional, es probable que su adición paralela al tratamiento convencional podría mejorar el pronóstico del paciente. Además, si bien el seguimiento de los dos casos fue durante seis meses, creemos necesario incrementar el seguimiento mínimo a un año, para mostrar una mayor fiabilidad en la respuesta terapéutica sostenida en el tiempo.

Como limitantes del presente reporte, se debe mencionar la falta de disponibilidad de la TOHC en los centros hospitalarios del Ministerio de Salud de nuestro país, su alto costo monetario y la ausencia de un seguro de salud que asuma los gastos de su aplicación. El costo de las 30 sesiones del THOC, en nuestro país, es alrededor de 1500 dólares americanos. Para la realización de nuestro estudio, fue necesario solicitar la colaboración de una institución privada, la cual brindó la THOC para ambos pacientes. Resaltamos la posibilidad que la mejoría clínica en ambos casos puede ser independiente de la aplicación de la TOHC. Por esta razón, son necesarios estudios con mayor nivel de evidencia para delimitar el beneficio específico de la TOHC en el tratamiento de la mucormicosis.

En conclusión, la MAC es una infección emergente durante el curso de la pandemia que presenta una importante morbimortalidad, siendo el manejo precoz fundamental para controlar la infección. La administración de la TOHC, como tratamiento coadyuvante podría acortar el tiempo de tratamiento y estancia hospitalaria, con menor exposición a la nefrotoxicidad y disminución de la morbilidad. Adicionalmente, resaltamos la necesidad de realizar nuevos estudios de investigación que ofrezcan mejor nivel de evidencia.
